# Frailty Nexus: Community of practice for frailty researchers and healthcare professionals

**DOI:** 10.1016/j.tjfa.2025.100074

**Published:** 2025-08-05

**Authors:** Benignus Logan, Adrienne Young, Kristiana Ludlow, David Ward, Leila Shafiee Hanjani, Natasha Reid, Ruth E Hubbard

**Affiliations:** a Australian Frailty Network, The University of Queensland, Brisbane, Australia; b Centre for Health Services Research, The University of Queensland, Brisbane, Australia; c Department of Medicine, Mater Hospital, Brisbane, Australia; d Internal Medicine Services, The Prince Charles Hospital, Brisbane, Australia; e Department of General and Geriatric Medicine, Princess Alexandra Hospital, Brisbane, Australia

**Keywords:** Capacity building, Knowledge translation, Social networking

## Abstract

**Background:**

There has been success in implementing frailty education for healthcare professionals, but there remains a need to improve the knowledge and skills of researchers and healthcare professionals to develop, implement and evaluate frailty-focused research.

**Objectives:**

This paper describes how the Australian Frailty Network developed and evaluated a virtual community of practice (VCOP), a proven model for fostering knowledge mobilisation, to support researchers and healthcare professionals in advancing frailty research and practice in Australia.

**Design:**

Mixed methods.

**Setting:**

Australian research and healthcare workplaces.

**Participants:**

Researchers and healthcare professionals.

**Measurements:**

A survey of prospective members sought to define the VCOP’s purpose, membership and structure. An evaluation was undertaken 18 months post-commencement, guided by the RE-AIM framework to assess reach, effectiveness, adoption, implementation and maintenance.

**Results:**

Fifty-five prospective members completed the initial survey. There was wide agreement from respondents to be inclusive in defining membership. The preferred purposes of the group included networking, opportunities to gain feedback, review frailty research, and knowledge and skill acquisition. In response, *Frailty Nexus* was launched, with three core components (‘Learning Link-Up’, online learning events; ‘Nexus News’, newsletter sharing learning and research opportunities; ‘Nexus Nook’, a library of shared resources). Membership totalled 618 from 81 organisations. Ninety-six percent of surveyed members expressed satisfaction with *Frailty Nexus*.

**Conclusions:**

*Frailty Nexus* is contributing to capacity building in multidisciplinary and translational frailty research. This VCOP could serve as a model that can be adapted by others to improve research outcomes and policy implementation.

## 1 Introduction

Notable progress has been made in developing and implementing frailty education, particularly in Britain and Canada [1,2]. These efforts aim to enhance healthcare professionals' understanding of the clinical significance of identifying frailty and the potential adverse outcomes associated with it. Gaining this knowledge not only equips healthcare professionals to deliver quality patient care, but also responds to their expressed interest in learning more about frailty [3]. Although there have been successful efforts in frailty education more broadly, significant gaps remain in translational frailty research. There is a shortage of researchers and healthcare professionals equipped to develop, implement and evaluate frailty-focused research [4]. This is particularly the case in Australia [3,5], and limits knowledge translation into practice and policy which is critical to further optimising the health and wellbeing of people living with frailty.

Establishing research collaborations, partnerships and forums for sharing knowledge is one approach to building capacity for knowledge mobilisation to address this need [6]. These initiatives are grounded in social learning systems, which recognise value in the social nature of learning [7]. Wenger and colleagues conceptualised this as a community of practice (COP), which they define as a group of individuals who share a common concern, set of challenges, or passion for a particular topic, and who enhance their knowledge and expertise in that area through ongoing interaction and collaboration [8].

Although COPs have historically been associated with business settings, they are increasingly recognised as applicable to healthcare disciplines, particularly as a tool for building capacity for research and knowledge mobilisation [6,[9], [10], [11], [12]]. Knowledge mobilisation seeks to improve the connections between research, policy, and practice [13]. COPs can take many forms with a variety of structures, styles and names [8]. They can benefit their members by building their confidence, giving a sense of belonging, facilitating access to expertise, and enhancing their knowledge and skills through continuous learning [8,11,12]. Technology can be an enabler, with virtual COPs (VCOP) well positioned to address professional isolation and more easily foster peer collaboration and mentoring [11,14]. They may also offer solutions to inequities in access to specialist knowledge and support due to geographical location, cost and workloads [15]. This is particularly relevant in Australia where a geographically-dispersed workforce of healthcare professionals means many can be isolated in rural and remote settings.

The Australian Frailty Network (AFN) was established in 2022 through a National Health and Medical Research Council (NHMRC) Medical Research Future Fund (MRFF) Dementia Ageing and Aged Care Mission grant [5]. The AFN is a national collaborative group of researchers, students, healthcare professionals, non-government organisations, consumers (i.e., patients, people with lived experience, and unpaid caregivers) and policymakers. A key strategic priority for the AFN was to establish a national VCOP to support early career researchers and healthcare professionals with an interest in frailty to build capacity for translational frailty research. We defined early career researchers as: HDR (Higher Degree Research) students, i.e., people completing a Doctorate or Masters of Philosophy; and, early career academics (ECA), i.e., people within 5 years of completing their Doctor of Philosophy. Healthcare professionals include doctors, nurses, pharmacists and allied health professionals (e.g., physiotherapists, occupational therapists and dietitians). Supporting ECAs and healthcare professionals with an interest with research was particularly pressing given the challenges this cohort faced as a result of the COVID-19 pandemic, including funding uncertainty and competing clinical demands [16].

The purpose of this paper is to: (1) describe the development of a VCOP for frailty researchers and healthcare professionals in Australia; and (2) evaluate its reach, effectiveness, adoption, implementation, and maintenance. By sharing our experience in Australia, it may serve as a framework for researchers and healthcare professionals across various disciplines, and countries, to develop their own VCOP aimed at achieving their specific translational research objectives.

## 2 Methods

**Fig. 1:**
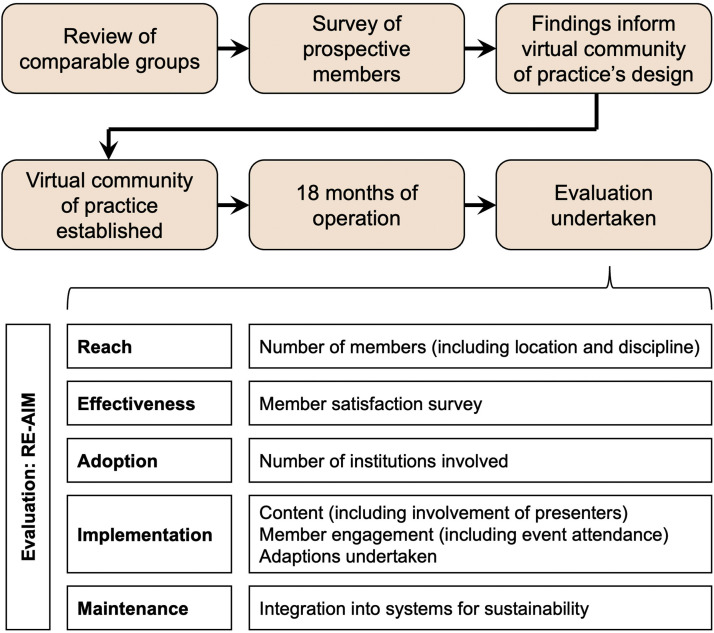
Overview of project’s approach.

The AFN formed a transdisciplinary working group to lead the development and evaluation of the proposed VCOP for HDR students, ECAs and healthcare professionals with an interest in frailty. The composition of the working group was intentionally diverse to ensure the VCOP would connect individuals across a range of disciplines. It included researchers and healthcare professionals with backgrounds in medicine, nursing, pharmacy and allied health professions. Fig. 1 provides an overview of the agreed approach for the project.

### 2.1 Development of the virtual community of practice

A review of existing comparable groups was undertaken as the first step in the process, given the scarcity of available literature about the development of VCOP in healthcare and academic settings. The purpose of this review was to gain an insight into their membership, purpose and operation, and what lessons they had learned from their functioning. A member of the team met with representatives from a total of eight groups across Australia, including The University of Queensland (UQ) Digital Health group, the Geriatric Clinical Academic Group of Sydney Health Partners, and the Better Evidence and Translation –Chronic Kidney Disease Post-Graduate and Early-Career Researcher group. The composition of these groups varied significantly - from a small, informal cluster of about 20 members based on a single university campus, which developed organically and maintained a largely social focus, to a national group of over a hundred members with formal terms of reference, a website, and an elected chairperson. We deliberately engaged with a diverse range of groups, so we could then match what our members needed with the different features identified in other groups.

These discussions with existing groups informed the design of an online survey of prospective members to identify their needs and preferences to inform decisions about the group’s membership, purpose, meeting frequency, and education topics. Convenience and snowball sampling was used to identify survey respondents. Invitations to complete an online questionnaire were emailed to all prospective members of the planned group (i.e., HDR students, ECAs, and healthcare professionals) identified by the researchers involved in establishing the AFN. Aligned with the AFN’s values of collaboration and inclusivity, we also sought broader participation by promoting the questionnaire through available social media channels of the AFN’s partner organisations and encouraging recipients of the email invitation to share it with other HDR students, ECAs, and healthcare professionals in their networks.

The questionnaire was designed specifically for this study by the research team and included both closed and open-ended questions (Supplementary File 1). It began with a short section on respondent's demographics. The main body of the questionnaire consisted of sections seeking respondent’s views on their current peer support, their preferences for the VCOP’s membership, purpose, content and logistics, and suggestions for the group’s name. The questionnaire was piloted on a small group of five early-career researchers to ensure clarity and validity. As a result of this process the term ‘peer support forum’ was used instead of ‘community of practice’ as this was identified as potentially being unfamiliar to some respondents. The questionnaire was administered in May 2023 on Qualtrics [17].

### 2.2 Evaluation of the virtual community of practice

The VCOP was designed by the working group based on findings from the review of comparable groups and online questionnaire (summarised in Fig. 1, and described in detail in the Results section). It was launched under the name “Frailty Nexus” in June of 2023. An evaluation was undertaken in November 2024 following the COP being operational for 18 months. This evaluation was underpinned by the RE-AIM (Reach, Effectiveness, Adoption, Implementation, Maintenance) framework [18] (Fig. 1). Data were collected via documents provided by the COP’s convenor (BL) and working group, and included metrics from the group’s Microsoft Teams site, YouTube, and Zoom accounts.

Reach was defined as the number of individuals who signed up as members, including their discipline as self-reported on their sign-up registration form. Adoption was assessed by the number of institutions involved, based on member’s email domains. Effectiveness was evaluated through a second online questionnaire, which was also administered via Qualtrics and included both closed and open-ended questions. It sought the respondent’s evaluation of the group’s purpose, learning events and communication activities, as well as their overall satisfaction (Supplementary File 1). It was distributed to all VCOP members in November 2024. Implementation measures included attendance at events (captured within the Microsoft Teams platform), views of YouTube recordings, number of email updates sent to members, and number of members requesting access to the VCOP’s Microsoft Teams site. Implementation measures also included who presented at events (topics and background of the presenters), the time needed for coordination, and the adaptions required in the operation of the VCOP. Maintenance was conceptualised as the extent to which the VCOP was embedded in systems to support its sustainability.

### 2.3 Data analysis

Descriptive statistics were used to summarise quantitative responses in both surveys, as well as the collected data from sign-up registration forms and metrics from Zoom, Microsoft Teams and YouTube. Categorical variables were expressed as numbers with valid percentages, with missing data excluded. Continuous variables were expressed as mean with standard deviation (SD) or median with interquartile range (IQR), depending on distribution characteristics. Analyses were undertaken in Microsoft Excel. No repeated measures analyses were undertaken for the two questionnaires as they contained different question sets. Thematic analysis of qualitative responses to open ended questions from the two surveys were planned, however this was not undertaken due to the low number of comments provided by respondents.

### 2.4 Ethical considerations

This research was approved by the UQ Human Research Ethics Committee (2023/HE000902). The consent process for both surveys was undertaken electronically via a Consent Statement. Sign-up registration data was accessed through an opt-out consent process.

## 3 RESULTS

### 3.1 Survey of prospective members

Fifty-five people completed the online questionnaire to inform the development of the VCOP. They were mostly from inner city locations (*n* = 27, 56 %) and mostly identified as being ECAs (*n* = 17, 23 %), doctors (*n* = 16, 22 %) or research students completing a Doctor of Philosophy (*n* = 14, 19 %) (Table 1). Whilst many were already satisfied with their current peer supports (*n* = 30, 63 %), most (*n* = 42, 84 %) expressed an interest in having more opportunities to interact with other researchers at a similar career stage and agreed that peer supports are beneficial (*n* = 44, 88 %) (Table 2).

**Table 1:** Characteristics of survey respondents.

Respondent Characteristic	Survey of prospective members n=55 (May 2023)	Evaluation survey n=61 (Nov 2024)
Location *		
Metropolitan – inner city	27 (56 %)	32 (52 %)
Metropolitan – suburban	14 (29 %)	15 (25 %)
Rural and remote	7 (15 %)	14 (23 %)
Role *†*		
Doctor of Philosophy student	14 (19 %)	9 (12 %)
Masters of Philosophy student	0 (0 %)	2 (3 %)
Early-career academic (ECA)	17 (23 %)	12 (15 %)
Clinician undertaking a research project	11 (15 %)	9 (12 %)
Clinician: doctor	16 (22 %)	15 (19 %)
Clinician: nurse	3 (4 %)	10 (13 %)
Clinician: allied health professional	9 (12 %)	17 (22 %)
Clinician: pharmacist	0 (0 %)	3 (4 %)
Health care student	3 (4 %)	0 ( %)
Other	0 (0 %)	1 (1 %)

Note: *Not all respondents answered this question; † Respondents were able to select multiple answers

**Table 2:** Survey of prospective members.

	Strongly Agree	Agree	Neutral	Disagree	Strongly Disagree
**Current peer supports**					
I have existing opportunities where I can interact with other researchers at a similar career stage *	9 (18 %)	23 (46 %)	8 (16 %)	9 (18 %)	1 (2 %)
I am satisfied with my current peer supports *†*	8 (17 %)	22 (46 %)	8 (17 %)	10 (21 %)	0 (0 %)
I think peer support networks are beneficial *	29 (58 %)	15 (30 %)	5 (10 %)	0 (0 %)	1 (2 %)
I would like more opportunities to interact with other researchers at a similar career stage *	24 (48 %)	18 (36 %)	5 (10 %)	2 (4 %)	1 (2 %)
I am comfortable reaching out to peers within my team for support *	14 (28 %)	24 (48 %)	7 (14 %)	4 (8 %)	1 (2 %)
I am comfortable reaching out to peers outside my team for support *	11 (22 %)	16 (32 %)	15 (30 %)	8 (16 %)	0 (0 %)
**Desired purpose for the group**					
Knowledge and skill acquisition (including masterclasses on research methods and transferable skills) *‡*	22 (50 %)	17 (39 %)	2 (5 %)	3 (7 %)	0 (0 %)
Networking (including identifying opportunities for collaboration with peers) *‡*	21 (48 %)	22 (50 %)	1 (2 %)	0 (0 %)	0 (0 %)
Opportunities to present research to peers (including preparation for milestone and conference presentations, and feedback on grant applications) *‡*	13 (30 %)	24 (55 %)	6 (14 %)	1 (2 %)	0 (0 %)
Opportunities to gain feedback from independent senior researchers (which may include ‘incubator sessions’ where a project plan is presented and feedback provided by an expert panel) *‡*	17 (39 %)	23 (52 %)	3 (7 %)	1 (2 %)	0 (0 %)
Review of developments in frailty research (similar approach to a journal club) *‡*	20 (45 %)	20 (45 %)	4 (9 %)	0 (0 %)	0 (0 %)
Social events and interactions *‡*	10 (23 %)	29 (43 %)	13 (30 %)	2 (5 %)	0 (0 %)
Library of shared resources *‡*	17 (39 %)	20 (45 %)	5 (11 %)	2 (5 %)	0 (0 %)

Note: *50 respondents; †48 respondents; ‡44 respondents. HDR: Higher Degree Research.

Respondents identified the top priorities for the VCOP’s purpose (as indicated by agreement and strong agreement responses, Table 2) to include: networking (*n* = 43, 98 %); opportunities to gain feedback from independent senior researchers (*n* = 40, 91 %); review of developments in frailty research (*n* = 40, 90 %); and, knowledge and skill acquisition (*n* = 39, 89 %). There was broad consensus that membership should be inclusive. “Frailty Nexus” was one of the nine names suggested by respondents for the group.

### 3.2 Virtual community of practice established

The VCOP was launched as “Frailty Nexus” in June 2023, with membership open to HDR students, ECAs, and healthcare professionals (doctors, nurses, pharmacists and allied health professionals) with an interest in frailty related research. To join the group, individuals completed a short online registration form. The group was promoted by AFN and its partner organisations through emails, social media posts, and at seminars and conferences.

The purpose for *Frailty Nexus* was articulated as:

- Networking (including identifying opportunities for collaboration with peers),
- Opportunities to gain feedback from independent senior researchers (which may include ‘incubator sessions’ where a project plan is presented to an expert panel),
- Review of developments in frailty research (similar approach to a journal club),
- Knowledge and skill acquisition (including masterclasses on research methods and transferable skills),
- Opportunities to present research to peers (including preparation for conference presentations, student milestones, and feedback on grant applications), and
- Library of shared resources (including HDR students sharing their milestone documents, and members sharing presentations they have written which may be of interest to others).

To achieve this purpose, three key components were ultimately developed. *Learning Link-Up* is a monthly online Zoom session which was organised to provide members with a forum for learning and networking. These sessions include a review of developments in frailty research, provide masterclasses on research methods, and offer the ability for peers to present to each other about their own research. *Nexus News* is a regular email newsletter which shares with members various learning and research opportunities which may be of interest to them, including access to funding and grants. *Nexus Nook* is a Microsoft Teams site established to provide a library of shared resources which members can access and contribute to. Presenters from *Learning Link-Ups* share relevant materials in this library for ongoing access by their peers. YouTube weblinks to past recordings are also made available on *Nexus Nook*.

### 3.3 Evaluation

In the first 18 months of operation, *Frailty Nexus* reached a total number of 618 registered members, representing diverse locations, roles and disciplines as indicated by the respondents of the evaluation survey (Table 1). Registration data demonstrated 384 (62 %) members had a primary role as a healthcare professional. This does not account for other academic affiliations they may hold. The healthcare professionals included 184 (30 %) doctors, 98 (16 %) nurses, 30 (5 %) physiotherapists, 24 (4 %) pharmacists, 23 (4 %) occupational therapists, and 17 (3 %) dietitians. Academics numbered 158 (26 %), which included 49 (8 %) HDR students and 41 (7 %) ECAs. Analysis of members’ email domains suggests that members were affiliated with 81 different organisations. Of these organisations, 34 (42 %) were educational institutions and 33 (41 %) were hospitals and health services, with the rest (*n* = 14, 17 %) a mix of government statutory bodies and community-based allied health or nursing home providers.

**Fig. 2:**
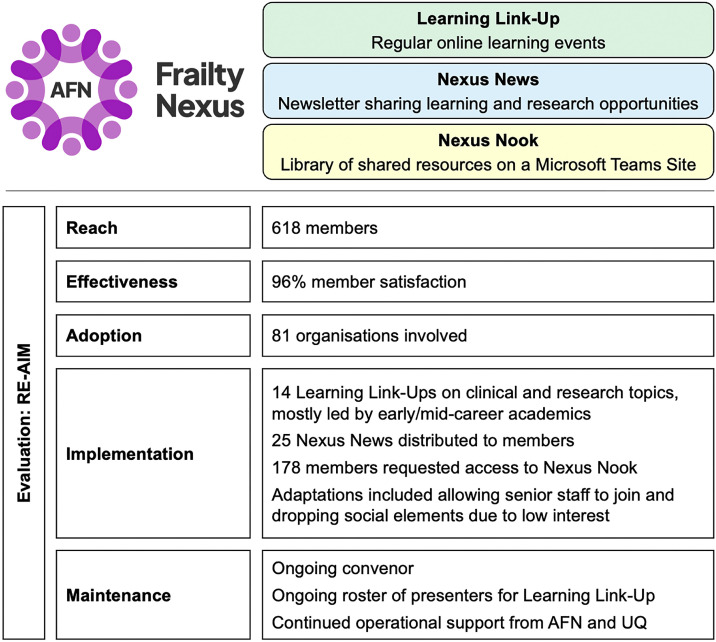
Overview of the evaluation of Frailty Nexus (AFN: Australian Frailty Network; UQ: University of Queensland).

All three components of *Frailty Nexus* were implemented, as summarised in Fig. 2, The VCOP was supported by a convenor who contributed an average of 6 h per month to coordinate *Frailty Nexus* (initially funded by the AFN). Fourteen *Learning Link-Ups* were held, as summarised in Table 3, with a mix of academic and clinical content. Fifteen presenters were involved: 2 HDR students; 10 early/mid-career academics; and 3 professors. There was a median of 51 (IQR: 40–59) live attendees at these events, and the recordings were later watched by a median of 55 (IQR: 44–76) attendees. *Nexus News* was sent to the membership a total of 25 times over the 18 months. Of the 618 members, 172 (27.8 %) requested access to *Nexus Nook*, the library of shared resources on a Microsoft Teams site.

**Table 3:** *Learning Link-Up*: topics, presenters and attendance.

Date	Event (Presenter)	Attendance	Recording views
July 2023	How to get that grant! (Dr Adrienne Young, Dr David Ward, Dr Kristiana Ludlow)	36	49
August 2023	An introduction to implementation science (Dr Adrienne Young)	25	63
September 2023	Behind the evidence - Spotlight on donanemab for Alzheimer's Disease (Dr David Ward)	27	49
October 2023	Masterclass – Fundamentals of qualitative research (Dr Kristiana Ludlow)	43	76
November 2023	How much is too little? (Dr Jackson Fyfe)	52	100
February 2024	A practical introduction to systematic reviews (Dr Natasha Reid)	85	81
March 2024	The power of infographics (Dr Michael Lawless)	60	55
May 2024	Optimising polypharmacy and deprescribing with digital health innovations (Dr Lisa Kouladjian O’Donnell)	45	67
June 2024	Person-centred care in geriatric emergency medicine (Dr James van Oppen)	85	139
July 2024	Tapping into subjectivity - an introduction to Q-methodology (Dr Kristiana Ludlow)	57	29
August 2024	Behind the evidence - A critical analysis of OUR research (Dr Emily Gordon, Dr David Ward)	49	30
September 2024	Strengths and frailty in older Aboriginal and Torres Strait Islander Peoples (Dr Ebony Lewis, Dr Jesse Zanker)	85	x *
October 2024	Succeeding in academic writing (Prof Ruth Hubbard, Prof Leon Flicker, Prof Mark Hughes)	57	44
December 2024	The Genetics of Frailty (Mr Jonny Flint)	39	30

Note: x * Video under embargo.

Sixty-one members completed the evaluation survey. There was broad agreement from members that *Frailty Nexus* was meeting its stated purpose, and most people felt the *Learning Link-Up* was beneficial to their learning and networking (Table 4). Fifty-seven respondents (96 %) agreed or strongly agreed that they were satisfied overall with Frailty Nexus.

**Table 4:** Evaluation of Frailty Nexus.

	Strongly Agree	Agree	Neutral	Disagree	Strongly Disagree
***I believe that Frailty Nexus has provided opportunities to…***					
Interact with other researchers and clinicians with a shared interest in frailty *†*	21 (36 %)	28 (47 %)	9 (15 %)	0 (0 %)	1 (2 %)
Gain feedback from independent senior researchers *†*	14 (24 %)	19 (32 %)	20 (34 %)	6 (10 %)	0 (0 %)
Learn about developments in frailty research *	32 (53 %)	26 (43 %)	1 (2 %)	0 (0 %)	1 (2 %)
Acquire new knowledge and skills *	26 (43 %)	30 (50 %)	4 (7 %)	0 (0 %)	0 (0 %)
Access a library of shared resources *†*	15 (25 %)	23 (39 %)	20 (34 %)	1 (2 %)	0 (0 %)
***I believe the ‘Learning Link-Up’ events each month…***					
Meets my learning needs *†*	13 (22 %)	41 (69 %)	4 (7 %)	1 (2 %)	0 (0 %)
Appropriately balances a mix of academic and clinical topics *†*	14 (24 %)	38 (64 %)	6 (10 %)	0 (0 %)	1 (2 %)
Provides adequate opportunities for interaction with peers *†*	12 (20 %)	28 (47 %)	18 (31 %)	1 (2 %)	0 (0 %)
Provides adequate opportunities to ask questions of the presenters *	14 (23 %)	38 (63 %)	8 (13 %)	0 (0 %)	0 (0 %)
***Overall, I am satisfied with Frailty Nexus****†*	29 (49 %)	28 (47 %)	2 (3 %)	0 (0 %)	0 (0 %)

Notes: *60 respondents; †59 respondents.

Several adaptions were made to Frailty Nexus during its implementation. Through the launch of *Frailty Nexus*, an intentionally inclusive approach to membership was adopted, with senior academics and healthcare professionals allowed to join as members. Initially intended as an early-career forum, it was not anticipated this forum would be of interest to them. *Learning Link-Ups* were not held every month as initially intended, to account for breaks during school holidays when many members are on leave. A planned *Learning Link-Up* featuring a Christmas Quiz with a more social focus was cancelled due to a lack of registrations. Initially a Whatsapp group was envisioned as a method of keeping members connected and interactive, but was abandoned as it gained no traction.

Maintenance of *Frailty Nexus* is evidenced at an individual and organisational level. At an individual level there have been ongoing requests by healthcare professionals and researchers to join. From the conclusion of the evaluation process in December 2024 through to the time of publication, the membership has continued to grow from 618 members to 853. Aside from the first few months of operation in 2023 there has been limited promotion undertaken with word-of-mouth recommendations and informal networks responsible for much of this growth. Only three requests have been made for removal from the group and its associated *Nexus News* mailing list. There has been sustained attendance at *Learning Link-Up* events since the formal evaluation concluded in 2024, with the most recent event in May 2025 having 74 attendees.

At an organisational level, *Frailty Nexus* has been well embedded within AFN. This is demonstrated in there being an ongoing convenor (member of the AFN team) who continues to coordinate the *Frailty Nexus*, with plans underway to transition this role to a new ECA. There is an ongoing roster of willing presenters for the *Learning Link-Up* who continue to contribute their time and expertise on an in-kind basis. The AFN Executive Leadership Team continues to oversee the *Frailty Nexus* with a commitment to its continuation, with support from the AFN team more broadly, and partnership with the University of Queensland who provides the necessary infrastructure, systems and IT services. The endeavours of *Frailty Nexus* are reported to the NHMRC as part of annual reporting required for the MRFF grant which enabled the establishment of AFN.

## 4 Discussion

This paper presents the development and evaluation of *Frailty Nexus*, a VCOP established for frailty researchers and healthcare professionals to help build capacity for translational frailty research in Australia. It details how its development was informed by the needs and preference of the target group, by surveying prospective members about the group's purpose and membership. Its initiatives, including *Learning Link-Up, Nexus News* and *Nexus Nook*, reached 618 people from at least 81 different organisations within the first 18 months of operation, and 96 % of surveyed members expressed satisfaction with *Frailty Nexus*.

The reasons for *Frailty Nexus* being established are comparable to those cited previously for the creation of other VCOPs. They included the need to address professional isolation, foster peer collaboration, and facilitate networking [14,15]. *Frailty Nexus* is particularly beneficial for researchers and health professionals who lack established peer networks to foster learning and develop informal COPs.

There is limited literature which explores what makes a COP successful, particularly in a healthcare discipline. Stoll and colleagues propose some key characteristics of what makes a professional learning community effective [19]. *Frailty Nexus* has achieved some of these, particularly having a shared vision of purpose developed at the outset by consultation with prospective members, and promoting collective responsibility for learning by having most of the *Learning Link-Up* events presented by HDRs or early/mid-career academics. The favourable member satisfaction expressed in the evaluation survey could be seen as reflective of a respectful and supportive environment. The VCOP has demonstrated inclusive membership, with efforts to look outside of immediate networks for sources of learning. This is evidenced by inviting mid-career academics from Britain and Canada to be speakers at *Learning Link-Ups*.

There are however some of Stoll et al.’s characteristics which are less evident in *Frailty Nexus* at this time. Reflective professional enquiry has not been embedded within the VCOP with no opportunities built into *Learning Link-Up* for reflecting on the learning as a collective group. There has also been minimal meaningful collaboration between members during the learning events, and in how *Nexus Nook* is being utilised as a shared library, with most content contributed by the convenor and presenters

In addition to the work from Stoll and colleagues, Pariser et al. [20] identified the involvement of members in the development of the group as an enabler to successful implementation of a COP. This is something which *Frailty Nexus* achieved. O’Donnell and colleagues argue that communities that have a clear, defined purpose with a high level of activity are more likely to be successful [21]. *Frailty Nexus* particularly benefitted from the time taken to articulate its purpose and did so with the input of prospective members. The regular *Learning Link-Ups* and *Nexus News* help generate regular activity to facilitate member engagement.

Longitudinal evaluation of 16 COPs by Antonacci et al. [22] demonstrated the need for centralised leadership structure and frequent rotation of convenorship over time to sustain and grow COPs. The AFN’s governance of the *Frailty Nexus* may prove to be an enabler for its sustainability, particularly through the implementation of strategies to support succession planning and capacity building for future convenors of the group.

Mentorship is recognised as an essential component of professional development and promotes academic productivity [23,24]. It helps ECAs understand expectations of the academic environment, and facilitates a sense of community [24,25]. A mentoring component was not included in the initial development of our VCOP, as it was determined to be beyond the original scope and resources available to the working group at the time. Now *Frailty Nexus* has been established, and enjoyed some initial success, the group is now able to consider implementing a development in this area to support members achieve their translational frailty research goals.

There are a range of possible structures to adopt, from traditional dyadic mentorship relationships, through to more informal peer mentoring [25]. Work from the Gerontological Society of America (GSA) offers a potential framework via their ‘Advancing Gerontology through Exceptional Scholarship’ mentoring initiative aimed at fostering productivity and peer support for ECAs [26]. The GSA set out a number of recommendations for an effective mentoring program, including finding the appropriate format, taking advantage of virtual engagement, ensuring representation across the career spectrum in planning, ensuring diversity among leadership, and developing mechanisms to align content, incorporate feedback and document success [24]. As an initial step to better supporting mentoring within *Frailty Nexus* we will seek to emulate the GSA’s “Career Conversations” sessions [24], with future *Learning Link-Ups* incorporating panel discussions about networking and developing mentoring relationships modelled from those used by the GSA [24].

The strength of this study lies in its documentation of an approach to developing, implementing, and evaluating a COP within the healthcare field, an area with limited existing literature. This could potentially act as a model for researchers and healthcare professionals in different healthcare disciplines, and other countries, to establish their own COP to address their identified translational research goals. Relevant considerations would include: investigating what comparable COP exist and understanding what lessons they have learnt in their operations; involving relevant stakeholders in the design of the COP; utilising technology to increase member connectedness; and ensuring evaluations are comprehensive, with the use of RE-AIM as a possible approach for this.

Many of this paper’s limitations relate to the quality of the evaluation measures utilised, including a reliance at this point in the VCOP’s lifecycle on self-reported measures. Firstly, the response rate from members in the evaluation survey was low, and likely reflects a degree of survey fatigue. This low response rate introduces the risk of non-response bias, so the reported member satisfaction should be interpreted with some caution. Furthermore, since demographic data relied on self-reported survey responses, the low response rate may limit the accuracy of our assessment of the VCOP’s representativeness. The low response itself may also indicate limited member engagement. A higher response rate might have allowed for more robust inferential analysis, which would have strengthened our analysis.

Secondly, our estimation of adoption, using email domains, is underreported as it does not account for the organisations of those 144 members who registered using a personal email domain (e.g., gmail.com), nor accounts for members who have more than one affiliation (i.e., clinical academics who have roles with a hospital and a university). Finally, qualitative methods such as collecting data through focus groups, could have provided richer insights into member's experiences and outcomes, but resource restraints made this unfeasible at this time. To enhance our understanding, future evaluations would benefit from the inclusion of such qualitative methods.

AFN’s original objective in establishing this VCOP was to strengthen capacity for translational frailty research. However, conducting an evaluation only 18 months after the launch of *Frailty Nexus* provides a limited window to fully assess its translational research outcomes. This is a notable limitation of this evaluation, as the benefits of communities of practice and capacity-building initiatives often take longer to emerge [27].

To gain a more comprehensive understanding of *Frailty Nexus*’ long-term effectiveness and impact, a longitudinal evaluation should be prioritised by AFN. This would provide the opportunity to explore research outputs and collaborations resulting from a member’s involvement with *Frailty Nexus*, and any practice changes which eventuated. To date we are not aware of any impacts of this nature. Furthermore, the long-term sustainability of this VCOP –as well as the strategies required to maintain, adapt, and evolve both the initiative and member engagement –remains uncertain and will need ongoing attention. More formal institutional or sustainability indicators would be beneficial in assessing this.

## 5 Conclusion

*Frailty Nexus* is a VCOP dedicated to helping researchers and healthcare professionals to develop, implement and evaluate frailty-focused research in Australia. This is supported by its initiatives including *Learning Link-Up, Nexus News*, and *Nexus Nook*. As a capacity building endeavour, it aims to ultimately benefit the health and wellbeing of people living with frailty by equipping researchers and healthcare professionals to translate frailty-focused research into policy and practice. Researchers and healthcare professionals in other settings may consider adopting the AFN’s approach to developing and implementing a VCOP to support the achievement of their own specific capacity building goals. Specifically we would recommend they explore comparable COPs to identify lessons learned from their operations, engage relevant stakeholders in the COP’s design, leverage technology to enhance member connectedness and equitable access, and conduct thorough evaluations using a method such as the RE-AIM framework to inform ongoing improvements. Future research is needed to demonstrate whether the group’s operations are sustained over a longer period and continues to meet the needs of its members, and what impact this has on members, their research and their clinical practice.

## Declarations

### Ethics approval and consent to participate

This research obtained ethical approval from the University of Queensland Human Research Ethics Committee (Reference: 2023/HE000902).

## Consent for publication

Not applicable.

## Availability of data and materials

The datasets used and/or analysed during the current study are available from the corresponding author on reasonable request.

## Authors' contributions

All authors were involved in this study’s design and concept.

REH is the Principal Chief Investigator of the MRFF grant, with AY and NR being Chief Investigators. BL is the inaugural convenor of Frailty Nexus.

BL completed the primary data analysis and interpretation.

AY conceptualised the evaluation approach.

BL led the writing of the manuscript, with input from all co-authors.

All authors contributed to revising the manuscript, and read and approved the final version.

## Funding

This research is funded by a 10.13039/501100000925National Health and Medical Research Council grant. Specifically, a Medical Research Future Fund (MRFF) Dementia, Ageing and Aged Care Mission grant, awarded in 2021 (APP2016045).

## Declaration of competing interest

The authors declare that they have no competing interests.

## Appendix Supplementary materials

Image, application 1
