# Supplementary material for: Frailty Nexus: Community of practice for frailty researchers and healthcare professionals
Source: J Frailty Aging. 2025 Aug 5;14(5):100074. doi: 10.1016/j.tjfa.2025.100074 (PMC12375211; doi:10.1016/j.tjfa.2025.100074)
Supplement: Supplementary file 1 [file mmc1.docx]

## Survey 1: Prior to establishment of the peer forum

## Introduction and Consent Statement

### Establishment of a peer forum for research students and early-career academics in frailty

Principal Investigators: Dr Benignus Logan, Dr Frederick Graham, Dr Bonnie Kwok, Dr Kristiana Ludlow, Dr Natasha Reid, Dr Leila Shafiee Hanjani, Dr David Ward, Dr Adrienne Young, Prof Ruth Hubbard

#### Context

The Australian Frailty Network (AFN) has been established by a Medical Research Future Fund (MRFF) grant from the National Health and Medical Research Council (NHMRC) (APP: 2016045) with the vision of delivering a national response to frailty and helping all Australians age well.

A key component of the work will be to support training to build capacity for translational frailty research. An initiative to achieve this is the establishment of a peer forum to bring together those research students and early-career academics in frailty to facilitate targeted training, upskilling, networking and coaching. Those who have research interests aligned to frailty, such as in aged care and geriatric medicine, are also welcome.

This research survey seeks to identify the preferences of potential members of this peer forum with regards to its membership, articulated purpose, meeting frequency, and education topics.

#### What we are seeking from you

We are inviting you to participate in this survey as you have been identified as a potential member of this peer forum due to your role as a:

- Higher Degree Research student (MPhil or PhD),
- Early-career academic, or
- Health care professional or student who is undertaking a relevant research project as part of their professional development.

Questions will seek your input on the design and operation of this peer forum.

The survey is being administered on the secure Qualtrics platform.

#### Further details on confidentiality and ethical principles

Participation in this study is voluntary. Completion of the survey will be interpreted as consent to participate. Data is deidentified. There is no way of withdrawing consent once the survey responses are submitted. You can withdraw from the study prior to submission by closing your browser.

The survey is anticipated to take 10 minutes of your time. There will be no reimbursement provided. There are no identified risks to your completion of this survey.

All responses are anonymous. Any identifiable information provided in qualitative responses will be deidentified before analysis and reporting. Results from the research are intended for publication in a peer-reviewed journals and to inform future stages of the project. In any publication, information will be provided in such a way that you cannot be identified.

Data will be stored on UQ’s secure cloud platform (UQRDM) for 15 years in accordance with section 601.2/C124 and 601.2/C125 of the Queensland State Archive University Sector Retention and Disposal Schedule. Only the research team will have access to these data.

If you would like to receive a summary of the research findings once they become available, please contact Dr Benignus Logan ([benignus.logan@uq.edu.au](mailto:benignus.logan@uq.edu.au)).

This study adheres to the Guidelines of the ethical review process of The University of Queensland and the National Statement on Ethical Conduct in Human Research. Whilst you are free to discuss your participation in this study with the researcher contactable on [benignus.logan@uq.edu.au](mailto:benignus.logan@uq.edu.au), if you would like to speak to an officer of the University not involved in the study, you may contact the Ethics Coordinator on 617 3365 3924 / 617 3443 1656 or email [humanethics@research.uq.edu.au](mailto:humanethics@research.uq.edu.au)

This study has been approved by the University of Queensland Human Research Ethics Committee
(Reference: 2023/HE000902).

#### Are you willing to participate in this survey?

YES NO

#### Key contacts

##### Clinical contact person

Name: Dr Benignus Logan
 Position: Medical Monitor (Clinical Academic: Geriatrician)
 Telephone: 0407 125 182
 Email: benignus.logan@uq.edu.au

##### Independent contact person

Contact: Ethics Coordinator
 Telephone: 07 3365 3924 / 07 3443 1656
 Email: humanethics@research.uq.edu.au

## Demographics

Please select all the descriptors which best describes your role in completing this survey:
 (multiple selections allowed; this relates to all your work and study positions)

 MPhil student

 PhD student

 Early-career academic

 Clinician undertaking a research project

 Clinician: doctor

 Clinician: nurse

 Clinician: allied health professional

 Clinician: pharmacist

 Health care student: medicine

 Health care student: nursing

 Health care student: pharmacy and allied health

 Other *free text _____________*

Please select ONE descriptor which best describes your primary work/study location:
 Metropolitan: Inner-city

 Metropolitan: Suburban

 Rural or remote (including regional centres)

Please select the state/territory in which you primarily work/study:

 Australian Capital Territory

 New South Wales

 Northern Territory

 Queensland

 South Australia

 Tasmania

 Victoria

 Western Australia

 New Zealand

## Current peer support network(s)

Please indicate your response to each of the following statements

|  | Strongly disagree | Disagree | Neutral | Agree | Strongly agree |
| --- | --- | --- | --- | --- | --- |
| I have existing opportunities where I can interact with other researchers at a similar career stage | 🞏 | 🞏 | 🞏 | 🞏 | 🞏 |
| I am satisfied with my current peer supports | 🞏 | 🞏 | 🞏 | 🞏 | 🞏 |
| I think peer support networks are beneficial | 🞏 | 🞏 | 🞏 | 🞏 | 🞏 |
| I would like more opportunities to interact with other researchers at a similar career stage | 🞏 | 🞏 | 🞏 | 🞏 | 🞏 |
| I am comfortable reaching out to peers within my team for support | 🞏 | 🞏 | 🞏 | 🞏 | 🞏 |
| I am comfortable reaching out to peers outside my team for support | 🞏 | 🞏 | 🞏 | 🞏 | 🞏 |

## Membership

Please indicate your preference on which groups should be eligible for membership in the frailty peer forum:

|  | Disagree | Neutral | Agree |
| --- | --- | --- | --- |
| MPhil students | 🞏 | 🞏 | 🞏 |
| PhD students | 🞏 | 🞏 | 🞏 |
| Early career academics (<10 years post-doc) | 🞏 | 🞏 | 🞏 |
| Health care professionals undertaking research | 🞏 | 🞏 | 🞏 |
| Health care students undertaking relevant research | 🞏 | 🞏 | 🞏 |
| Advanced trainees  (doctors undertaking their specialty training) | 🞏 | 🞏 | 🞏 |
| Honours students | 🞏 | 🞏 | 🞏 |

If you wish to make any comments on the forum’s **membership**, please write them here:

__________________________________________________________________________________________________________________________________________________________________________________________________________________________________________________________________

## Purpose

Please indicate your preference of what the purpose of this forum should include (there can be multiple):

|  | Strongly disagree | Disagree | Neutral | Agree | Strongly agree |
| --- | --- | --- | --- | --- | --- |
| Knowledge and skill acquisition (including masterclasses on research methods and transferable skills) | 🞏 | 🞏 | 🞏 | 🞏 | 🞏 |
| Networking  (including identifying opportunities for collaboration with peers) | 🞏 | 🞏 | 🞏 | 🞏 | 🞏 |
| Opportunities to present research to peers (including preparation for milestone and conference presentations, and feedback on grant applications) | 🞏 | 🞏 | 🞏 | 🞏 | 🞏 |
| Opportunities to gain feedback from independent senior researchers (which may include ‘incubator sessions’ where a project plan is presented and feedback provided by an expert panel) | 🞏 | 🞏 | 🞏 | 🞏 | 🞏 |
| Review of developments in frailty research (similar approach to a journal club) | 🞏 | 🞏 | 🞏 | 🞏 | 🞏 |
| Social events and interactions | 🞏 | 🞏 | 🞏 | 🞏 | 🞏 |
| Library of shared resources (including HDR students sharing their milestone documents, and members sharing presentations they have written which may be of interest to others) | 🞏 | 🞏 | 🞏 | 🞏 | 🞏 |

If you wish to make any comments on the forum’s **purpose**, please write them here:

__________________________________________________________________________________________________________________________________________________________________________________________________________________________________________________________________

## Content

If there is consensus that the forum’s purpose should include knowledge and skill acquisition, a program of learning will be curated. Listed below are possible topics for inclusion. Kindly prioritise those topics of most interest by assigning a ranking of 1 to 14.

 Engaging with consumers

 Health economics

 Co-design

 Implementation science

 Qualitative research methodology

 Quantitative research methodology

 Pharmaco-epidemiology

 Behavioural psychology

 Biostatistics

 Presenting with impact

 Navigating grant writing and applications

 Supervising research

 Conducting systematic reviews

 Translational research

If there are any topics you feel should be considered for inclusion that are not listed in the above, please note them here:

__________________________________________________________________________________________________________________________________________________________________________________________________________________________________________________________________

## Logistics

Please indicate how frequently you would like the proposed peer forum to meet:
(as this is a national forum, they will occur virtually via Zoom or Teams)

 Weekly

 Fortnightly

 Monthly

Please indicate your availability to attend the forum:
(select all that apply – multiple options allowed)

 Start of the workday

 Mid-morning

 Lunch

 Mid afternoon

 End of the workday

 Outside of standard work hours (i.e. before or after work)

Please indicate if you would be interested in a communication avenue for discussions with peers:

 WhatsApp

 ‘Team’ in MS Teams

 Not interested

## Name of forum

If you have any suggestions on what to call this forum, please note them here (creativity welcomed):

____________________________________________________________________________________________________________________________________________________________________________

## Closing

If there are any comments or thoughts you wish to share but have not had the opportunity to as yet, please use the space below:

____________________________________________________________________________________________________________________________________________________________________________

***Thank you for your completion of this survey.***

## Survey 2: 18 months after the peer forum begins operation

## Introduction and Consent Statement

### Evaluation of Frailty Nexus by its members

Principal Investigators: Dr Benignus Logan, Dr Frederick Graham, Dr Bonnie Kwok, Dr Kristiana Ludlow, Dr Natasha Reid, Dr Leila Shafiee Hanjani, Dr David Ward, Dr Adrienne Young, Prof Ruth Hubbard

#### Context

The Frailty Nexus was established in June 2023 by the Australian Frailty Network (AFN), with the intent of building capacity for translational frailty research.

Now that Frailty Nexus has been operational for over 12 months, this research survey seeks to evaluate the satisfaction of Frailty Nexus members and understand how it can be modified to best meet their needs.

#### What we are seeking from you

We are inviting you to participate in this survey as you registered to be a member of Frailty Nexus and be placed on the group’s mailing list.

Questions will seek your opinions on what is working well, and what could be revised to make it more effective and beneficial for those involved.

The survey is being administered on the secure Qualtrics platform.

#### Further details on confidentiality and ethical principles

Participation in this study is voluntary. Completion of the survey will be interpreted as consent to participate. Data is deidentified. There is no way of withdrawing consent once the survey responses are submitted. You can withdraw from the study prior to submission by closing your browser.

The survey is anticipated to take 5-10 minutes of your time. There will be no reimbursement provided. There are no identified risks to your completion of this survey.

All responses are anonymous. Any identifiable information provided in qualitative responses will be deidentified before analysis and reporting. Results from the research are intended for publication in a peer-reviewed journals and to inform future stages of the project. In any publication, information will be provided in such a way that you cannot be identified.

Data will be stored on UQ’s secure cloud platform (UQRDM) for 15 years in accordance with section 601.2/C124 and 601.2/C125 of the Queensland State Archive University Sector Retention and Disposal Schedule. Only the research team will have access to these data.

If you would like to receive a summary of the research findings once they become available, please contact Dr Benignus Logan ([benignus.logan@uq.edu.au](mailto:benignus.logan@uq.edu.au)).

This study adheres to the Guidelines of the ethical review process of The University of Queensland and the National Statement on Ethical Conduct in Human Research. Whilst you are free to discuss your participation in this study with the researcher contactable on [benignus.logan@uq.edu.au](mailto:benignus.logan@uq.edu.au), if you would like to speak to an officer of the University not involved in the study, you may contact the Ethics Coordinator on 617 3365 3924 / 617 3443 1656 or email [humanethics@research.uq.edu.au](mailto:humanethics@research.uq.edu.au)

This study has been approved by the University of Queensland Human Research Ethics Committee (Reference: 2023/HE000902).

#### Key contacts

##### Clinical contact person

Name: Dr Benignus Logan
 Position: Medical Monitor (Clinical Academic: Geriatrician)
 Telephone: 0407 125 182
 Email: benignus.logan@uq.edu.au

##### Independent contact person

Contact: Ethics Coordinator
 Telephone: 07 3365 3924 / 07 3443 1656
 Email: humanethics@research.uq.edu.au

#### Are you willing to participate in this survey?

YES NO

## Demographics

Please select **ALL** the descriptors which best describes your role in completing this survey:
 (**multiple selections allowed**; this relates to all your academic and clinical positions)

 MPhil student

 PhD student

 Early-career academic

 Clinician undertaking a research project

 Clinician: doctor

 Clinician: nurse

 Clinician: allied health professional

 Clinician: pharmacist

 Health care student: medicine

 Health care student: nursing

 Health care student: pharmacy and allied health

 Other *free text _____________*

Please select **ONE** descriptor which best describes your primary work/study location:
 Metropolitan: Inner-city

 Metropolitan: Suburban

 Rural or remote (including regional centres)

Please select **ONE** location which best describes your primary work/study location:

 Australian Capital Territory

 New South Wales

 Northern Territory

 Queensland

 South Australia

 Tasmania

 Victoria

 Western Australia

 New Zealand

 Outside Australia and New Zealand

## Evaluation questions – purpose

Please indicate your level of agreement with each of the following statements.

**I believe that Frailty Nexus has provided opportunities to…**

|  | Strongly disagree | Disagree | Neutral | Agree | Strongly agree |
| --- | --- | --- | --- | --- | --- |
| - interact with other researchers and clinicians with a shared interest in frailty | 🞏 | 🞏 | 🞏 | 🞏 | 🞏 |
| - gain feedback from independent senior researchers | 🞏 | 🞏 | 🞏 | 🞏 | 🞏 |
| - learn about developments in frailty research | 🞏 | 🞏 | 🞏 | 🞏 | 🞏 |
| - acquire new knowledge and skills | 🞏 | 🞏 | 🞏 | 🞏 | 🞏 |
| - access a library of shared resources | 🞏 | 🞏 | 🞏 | 🞏 | 🞏 |

If you wish to make any comments about the forum’s **purpose**, please write them here:
(include what is working well, and what needs revision)

____________________________________________________________________________________________________________________________________________________________________________

## Evaluation questions – learning events

Please indicate your level of agreement with each of the following statements.

**I believe the ‘Learning Link-Up’ events each month…**

|  | Strongly disagree | Disagree | Neutral | Agree | Strongly agree |
| --- | --- | --- | --- | --- | --- |
| - meets my learning needs | 🞏 | 🞏 | 🞏 | 🞏 | 🞏 |
| - appropriately balances a mix of academic and clinical topics | 🞏 | 🞏 | 🞏 | 🞏 | 🞏 |
| - provides adequate opportunities for interaction with peers | 🞏 | 🞏 | 🞏 | 🞏 | 🞏 |
| - provides adequate opportunities to ask questions of the presenters | 🞏 | 🞏 | 🞏 | 🞏 | 🞏 |

If you wish to make any comments about the **Learning Link-Up program**, please write them here:

(include what is working well, what needs revision, and what future topics you would like covered)

____________________________________________________________________________________________________________________________________________________________________________

## Evaluation questions – communication preferences

Please indicate your level of agreement with each of the following statements.

**I believe the emails (‘Nexus News’) from Frailty Nexus…**

|  | Strongly disagree | Disagree | Neutral | Agree | Strongly agree |
| --- | --- | --- | --- | --- | --- |
| - are of an appropriate frequency | 🞏 | 🞏 | 🞏 | 🞏 | 🞏 |
| - are of interest to me | 🞏 | 🞏 | 🞏 | 🞏 | 🞏 |
| - adequately promote learning and other opportunities in the frailty discipline | 🞏 | 🞏 | 🞏 | 🞏 | 🞏 |

If you wish to make any comments about the **communication received from Frailty Nexus**, please write them here: (include what is working well, what needs revision)

____________________________________________________________________________________________________________________________________________________________________________

## Closing

Please indicate your level of agreement with the following statement.

|  | Strongly disagree | Disagree | Neutral | Agree | Strongly agree |
| --- | --- | --- | --- | --- | --- |
| Overall, I am satisfied with Frailty Nexus | 🞏 | 🞏 | 🞏 | 🞏 | 🞏 |

If there are any comments or thoughts you wish to share but have not had the opportunity to as yet, please use the space below:

____________________________________________________________________________________________________________________________________________________________________________

***Thank you for your completion of this survey.***
